# Rainfall Shapes the Diversity of Soil Nitrogen‐Fixing Microorganisms Worldwide

**DOI:** 10.1002/advs.77215

**Published:** 2026-08-17

**Authors:** Bin Hua, Shuang Pang, Ang Li, Zonghao Hu, Honghui Wu, Shuhan Zhang, Yi Fan, Yi Wu, Wei Yang, Yachao Zhao, Yupeng Guan, Baoming Ji, Deliang Kong, Yong Zhao, Anton A. Goncharov, Anastasia Yu. Korotkevich, Rong Mao, Yang Zhang, Ximei Zhang

**Affiliations:** ^1^ Experimental Centre of Forestry in North China Chinese Academy of Forestry Beijing China; ^2^ College of Forestry Henan Agricultural University Zhengzhou China; ^3^ Key Laboratory of Vegetation and Environmental Change Institute of Botany Chinese Academy of Sciences Beijing China; ^4^ Key Laboratory of Dryland Agriculture Institute of Environment and Sustainable Development in Agriculture Ministry of Agriculture Chinese Academy of Agricultural Sciences Beijing China; ^5^ State Key Laboratory of Efficient Utilization of Agricultural Water Resources CAU/CAAS Beijing China; ^6^ State Key Laboratory of Efficient Utilization of Arid and Semi‐arid Arable Land in Northern China Institute of Agricultural Resources and Regional Planning Chinese Academy of Agricultural Sciences Beijing China; ^7^ Key Laboratory of Arable Land Quality Monitoring and Evaluation Ministry of Agriculture and Rural Affairs Institute of Agricultural Resources and Regional Planning Chinese Academy of Agricultural Sciences Beijing China; ^8^ School of Grassland Science Beijing Forestry University Beijing China; ^9^ A.N. Severtsov Institute of Ecology and Evolution Russian Academy of Sciences Moscow Russia; ^10^ College of Forestry Jiangxi Agricultural University Nanchang China

**Keywords:** climate change, diazotrophic community, drought, *nifH*, nitrogen cycling, nitrogen fixation, precipitation increase, stochastic process

## Abstract

Soil nitrogen‐fixing microorganisms naturally fertilize terrestrial ecosystems, but the primary driver of their diversity across the globe and the underlying mechanisms remain unclear. We analyzed the *nifH* gene in 1257 (1137 publicly available + 120 self‐generated) soil metagenomes from 318 terrestrial ecosystems globally. Mean annual precipitation was identified as the key factor influencing the relative abundance, richness, and composition of the potential nitrogen‐fixers. Precipitation was directly associated with nitrogen‐fixers (e.g., water availability) rather than indirectly via other soil variables (e.g., pH). Lower precipitation increased the contribution of deterministic processes (e.g., interspecific competition) in driving their community assembly and selected species with larger genomes, while higher precipitation increased the contribution of stochastic processes (e.g., random birth/death) and favored smaller‐genome species. A multifactorial experiment further demonstrated that precipitation increase had a larger regulatory effect on the stochastic processes than other factors (e.g., climate warming). eXtreme Gradient Boosting (XGBoost) projections under future global change scenarios indicate a general increase in their relative abundance across most regions worldwide, with declines only in specific areas. These findings reveal distinct patterns and mechanisms governing the global biodiversity and biogeography of soil nitrogen‐fixers, providing valuable insights for developing region‐specific management strategies aimed at maintaining ecosystem productivity.

## Graphical abstract

This study reveals the distinctive pattern and mechanism of rainfall driving the global biodiversity and biogeography of soil potential N‐fixing microorganisms, and constructs the theoretical framework. The results guide us on how to maintain ecosystem productivity under future climate changes (e.g., whether a specific region needs more N fertilizers, N‐fixing agents, or other nutrients).

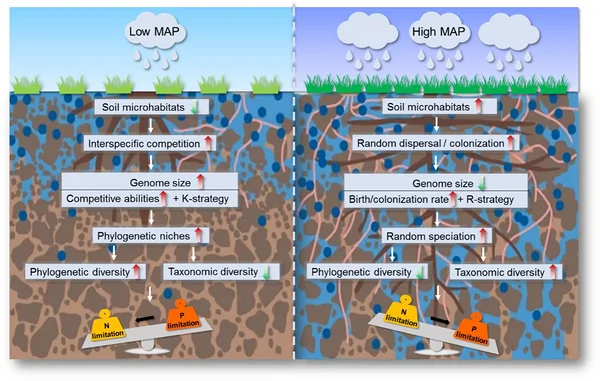

## 1 Introduction

Nitrogen (N) is the most essential element for all organisms, accounting for about 3% of their weight. The microorganism‐driven N fixation process can transform N_2_ into biologically available compounds at a rate of up to 100 Tg N per year, thus profoundly influencing the evolution and distribution of various organisms as well as the biodiversity and functions of various terrestrial ecosystems [1, 2, 3, 4]. A growing body of research has examined the diversity and composition of soil N‐fixing microbial communities across diverse ecosystems, including croplands, forests, and grasslands [5, 6, 7, 8, 9]. They have demonstrated that the diversity and composition of these communities were influenced by various factors such as agricultural management regime, soil physicochemical properties, and plant‐related parameters [10, 11, 12, 13, 14]. Most of these studies were conducted on relatively small spatial scales. The large‐scale studies were only a few, which found that the distribution of soil N‐fixing microorganisms was driven more by climatic factors than by soil properties [15, 16]. Actually, many climatic factors, especially precipitation, temperature, and aridity, are the key factors influencing the distribution of soil microorganisms in some cases [15, 16, 17]. However, a widely accepted and consistent conclusion on the most critical factor affecting soil N‐fixing microbial communities globally has still not been reached. Identifying the critical factor and revealing the underlying mechanism can help us understand how the soil N‐fixing microbiome and the associated N‐supply function respond to anthropogenic climate change.

We hypothesize that mean annual precipitation (MAP) is the most crucial factor driving the assembly of soil N‐fixing microbial communities globally; in other words, MAP should be more important than other climatic factors such as mean annual temperature, for four reasons. First, although soil pH rather than MAP is often identified as the crucial factor structuring bacterial communities on large spatial scales [18], microorganisms may acquire the N‐fixing capacity through horizontal gene transfer. Therefore, the primary driver for the specific functional microbial community may be different from that for the entire soil bacterial community. Second, water constitutes 60%–95% of the weight of all organisms, but MAP varies globally from extremely low to very high, emphasizing its potential vital role in structuring the N‐fixing microbiome. Third, MAP has already been found to be the most crucial factor driving the assembly of plant communities globally, followed by mean annual temperature, and then soil N availability [19]. Thus, it may be equally crucial for the soil N‐fixing microbial communities. Fourth, soil biodiversity and ecosystem functions have been found to be mainly governed by water availability in recent studies [20]. We further hypothesize that MAP governs the soil N‐fixing microbial community chiefly because it directly modulates the soil water supply instead of acting indirectly through other variables (e.g., additional soil physicochemical parameters and plant‐derived carbon), and because it exerts a distinctive control over the deterministic (e.g., interspecific competition and environmental filtering) vs. stochastic processes (e.g., random birth/death, species dispersal, speciation, and species extinction) that shape community assembly [21]. Specifically, soil microorganisms often survive in the microhabitats containing water, such as the water film layer surrounding soil particles or plant roots [22]. Low precipitation results in a reduced number of effective microhabitats, thereby intensifying interspecific competition. Consequently, larger‐genome species with stronger competitive abilities may outcompete smaller‐genome species [23]. In contrast, high precipitation increases the number of effective microhabitats, thereby promoting the random dispersal and colonization of species [24]. Thus, the smaller‐genome species are favored because they generally have higher birth rates and colonization rates compared with the larger‐genome species [23, 25].

This study aimed to advance the understanding of how climatic factors influence the potential N‐fixing microbiome by combining a global‐scale survey with a multifactorial field experiment (Figure 1). The survey combined 1137 publicly available soil metagenomes from 288 terrestrial ecosystems globally with 120 self‐collected soil metagenomes from 30 terrestrial ecosystems across China, which together belonged to 23 ecosystem types (Figure 1 and Table S1). The *nifH* gene responsible for potential N fixation in the metagenomes of the global‐scale survey (Table S2) and the multifactorial experiment (Table S3) was analyzed to (1) investigate whether MAP was the most critical factor influencing the potential N‐fixing microbial community across the globe; (2) examine whether MAP impacted the N‐fixing microbiome primarily via a direct route (i.e., changing the water resource) or indirect routes (e.g., altering other variables); (3) verify whether low MAP influenced the microbiome through increasing the contribution of deterministic processes whereas high MAP influenced it by increasing the contribution of stochastic processes; (4) test whether low MAP selected the potential N‐fixing microbial species with larger genomes whereas high MAP favored those with smaller genomes; (5) further examine whether the precipitation increase had a larger regulatory effect on the stochastic processes compared with other factors in driving the assembly of the N‐fixing microbial community in the multifactorial experiment; and (6) estimate how the relative abundance of soil potential N‐fixing microbiome would respond to the projected climate changes for every region on the Earth. Our results may reveal the pattern and mechanism of the global biodiversity and biogeography of soil nitrogen‐fixing microorganisms, thus guiding us to maintain the N‐supply function and ecosystem productivity under climate change.

**FIGURE 1:**
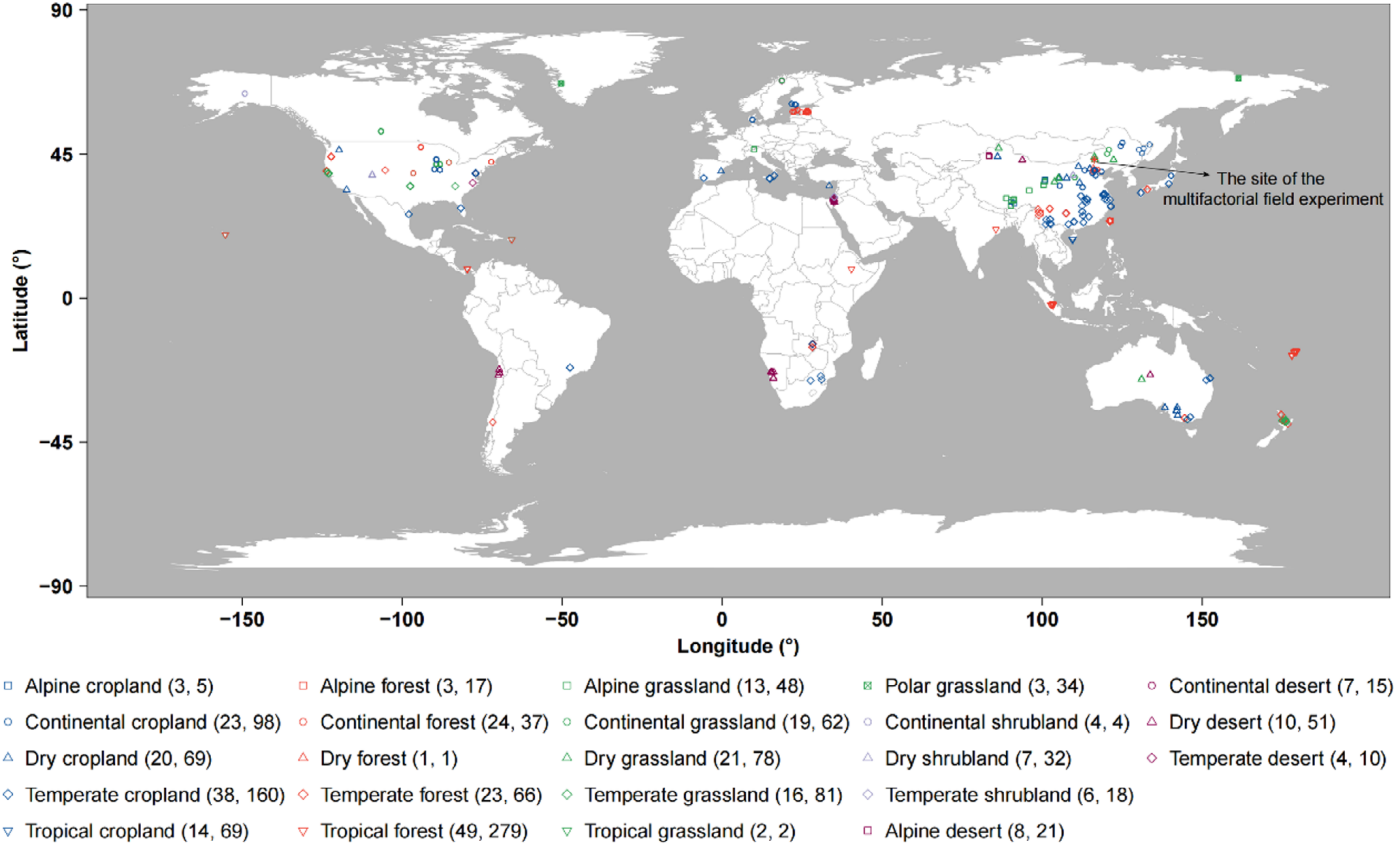
Locations (*n* = 318) of soil metagenomes (*n* = 1257) used in this study. These samples represented 23 distinct ecosystem types across the globe. The numbers within parentheses following each ecosystem type represent its location number and sample number, respectively.

## 2 Results and Discussion

This study revealed the distinctive pattern and mechanism of rainfall driving the global biodiversity and biogeography of soil potential N‐fixing microorganisms. Low rainfall reduced the number of soil microhabitats, intensified interspecific competition, selected the larger‐genome species, and decreased taxonomic diversity, suggesting that soil N limitation might become more severe and emphasizing the need for applying N fertilizer or N‐fixing agents. In contrast, high MAP increased the number of soil microhabitats, promoted the random dispersal/colonization of species, favored the smaller‐genome species, and increased taxonomic diversity, indicating that soil N limitation might be relaxed but other elements (e.g., phosphorus and trace elements) might be more limiting. Overall, our results guided us on how to maintain ecosystem productivity under future climate changes (e.g., whether a specific region needs more N fertilizers, N‐fixing agents, or other nutrients).

### 2.1 MAP as the Primary Driver of the Relative Abundance, Richness, and Composition of the Potential N‐Fixing Microbial Community on the Global Scale

A total of 10.136 ± 0.313 G (mean ± standard error) of metagenomic reads were detected for each of the 1257 soil samples, each of them containing at least 40 reads of the *nifH* gene (Table S2). The proportion of *nifH* reads to the total clean reads of each metagenome was used to represent its relative abundance. The relative abundance was 1.104 × 10^−5^ ± 3.383 × 10^−7^ (mean ± standard error) across all these samples. The Kruskal–Wallis test detected significant variation in the relative abundance across these ecosystem types (*p* < 0.001; Figure S1a). The mixed linear model showed that, of all climate, plant, and soil physicochemical variables considered, MAP exerted the strongest influence on the relative abundance (Figure 2a), and a significant positive linear correlation was observed between the relative abundance and MAP (*p* < 0.001; Figure 2b).

**FIGURE 2:**
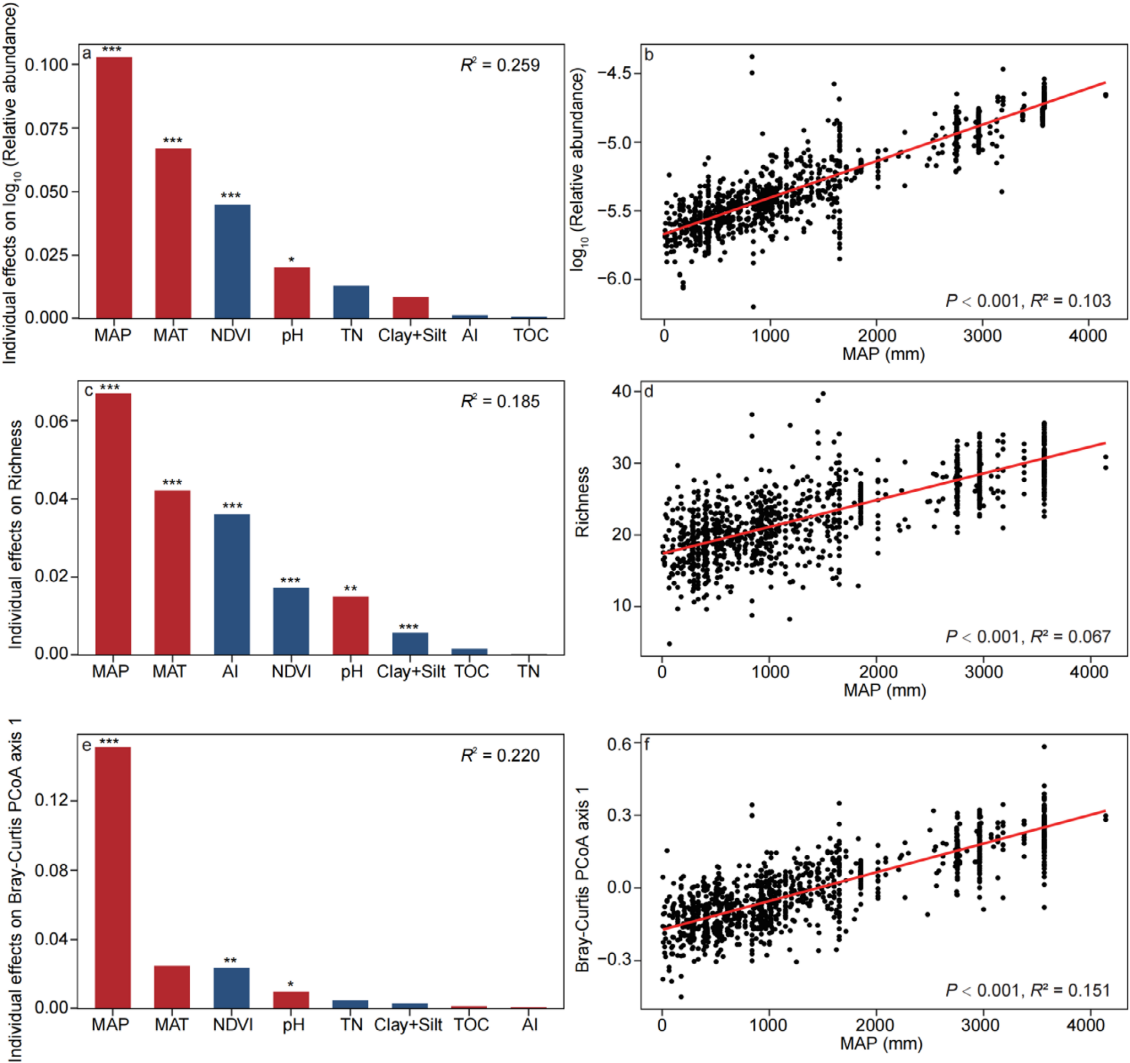
Contribution of various climate/plant/soil factors to the difference in the relative abundance (a), richness (c), and PCoA axis 1 (e) of potential N‐fixing microbial communities across different ecosystems, and the linear correlations between the three community variables and MAP (b,d, and f). In (a), (c), and (e), red and blue represent positive and negative effects, respectively; ^*^, ^**^, and ^***^ represent *p* < 0.05, 0.01, and 0.001, respectively. AI, Aridity index; Clay + Silt, clay and silt contents in soil; MAP, mean annual precipitation; MAT, mean annual temperature; NDVI, normalized difference vegetation index; TOC, soil total organic carbon content; TN, soil total nitrogen content.

For each sample, 40 *nifH* reads were randomly drawn to map to the full‐length reference sequences so as to estimate the richness of taxonomic operational units (OTUs). The richness was 23.407 ± 0.220 (mean ± standard error) across all these samples. Kruskal‐Wallis test also revealed significant differences in richness among these ecosystem types (*p* < 0.001; Figure S1b). Similar to the relative abundance, MAP was also identified as the most critical factor influencing richness (Figure 2c), and a significant positive linear correlation was noted between richness and MAP (*p* < 0.001; Figure 2d).

The permutational multivariate analysis of variance (PERMANOVA) detected significant variation in the OTU composition across these ecosystem types (*p* = 0.001; Figure S1c). The mixed linear model also showed that MAP was the most critical factor influencing the OTU composition (Figure 2e), and a significant linear correlation was observed between the principal coordinate analysis (PCoA) axis 1 and MAP (*p* < 0.05; Figure 2f). Consistently, the Mantel test showed that, among various environmental indices, MAP had the closest association with the composition of the potential N‐fixing microbial community (Table S4). The distance‐based redundancy analysis (dbRDA) also revealed that, among various factors, MAP had the largest effect on the OTU composition of the potential N‐fixing community in soil (Figure S2a). Actually, higher MAP increased the relative abundance of the potential N‐fixing microorganisms belonging to the first and fourth dominant phyla (Pseudomonadota and Themodesulfobacteria; Figure S3), reduced the abundance of those belonging to the second and third dominant phyla (Actinomycetota and Cyanobacteriota), but did not affect the abundance of those belonging to the fifth and sixth dominant phyla (Euryarchaeota and Gemmatimonadota).

The 40 reads of the *nifH* gene may not fully represent a soil N‐fixing microbial community. Therefore, we further analyzed only the 576 samples, each of which contained at least 200 reads. The rarefaction curve becomes relatively flat at this depth (Figure S4), indicating that this depth can effectively reveal the differences in the diversity and composition of N‐fixing microorganisms across different samples. MAP continued to emerge as the predominant factor governing the relative abundance, richness, and community composition of the N‐fixing microbial community at the global scale (Figure S5).

In addition, the limited number of soil physicochemical indices downloaded for the publicly available soil metagenomes may lead to an underestimation of their significance and thus an overestimation of the role of climatic factors in shaping the potential N‐fixing microbial community. Thus, we further analyzed only the 120 samples collected across China by ourselves, each of which had detailed information on various soil physicochemical indices. MAP continued to emerge as the predominant factor governing the relative abundance, richness, and community composition of the N‐fixing microbial community (Figure S6).

Furthermore, we considered the phylogenetic relationships among these reads to assess the diversity and composition of these communities. Consistently, the Kruskal–Wallis test and PERMANOVA revealed significant differences in the phylogenetic diversity and composition among these ecosystem types, respectively (*p* < 0.001; Figure S7). MAP likewise emerged as the dominant determinant shaping both the phylogenetic diversity and composition (Figure S8). Moreover, dbRDA also revealed that, among various factors, MAP had the largest effect on the phylogenetic composition of the soil potential N‐fixing community (Figure S2b). However, the linear relationship between the phylogenetic diversity and MAP was negative (Figure S8b), which was contrary to the positive correlation between the OTU richness and MAP (Figure 2d). The opposite responses of taxonomic and phylogenetic diversity to MAP were because the communities with higher taxonomic diversity consisted of more closely related species. In contrast, the species with lower taxonomic diversity comprised more distantly related species. Only a few previous studies discovered this interesting phenomenon in soil microbial communities [26].

### 2.2 MAP Influenced the Potential N‐Fixing Microbial Community Primarily via Direct Rather Than Indirect Routes

Structural equation modeling (SEM) was used to assess how strongly the climatic factors acted through direct vs. indirect pathways to shape the relative abundance, richness, and composition of the soil N‐fixing microbial community (Table S5). The final SEM provided an adequate fit to the data, capturing how MAP and mean annual temperature (MAT) shaped the N‐fixing microbial community (Fisher's *C* = 30.871, *df* = 26, *p* = 0.233, *n* = 1253; Figure 3a and Table S6). The model accounted for 23%, 12%, and 21% of the variance in microbial community relative abundance, richness, and composition, respectively. MAP exerted a strong direct influence on the community; the direct effects of MAP, expressed as standardized path coefficients, were 0.595, 0.458, and 0.618 for relative abundance, richness, and composition, respectively. Consistently, the direct effect was always larger than the indirect effect for all three community variables (Figure 3b), demonstrating that MAP influenced the potential N‐fixing microbial community primarily through its direct effect on the water resource rather than its indirect effect on other variables (e.g., other soil physicochemical indices).

**FIGURE 3:**
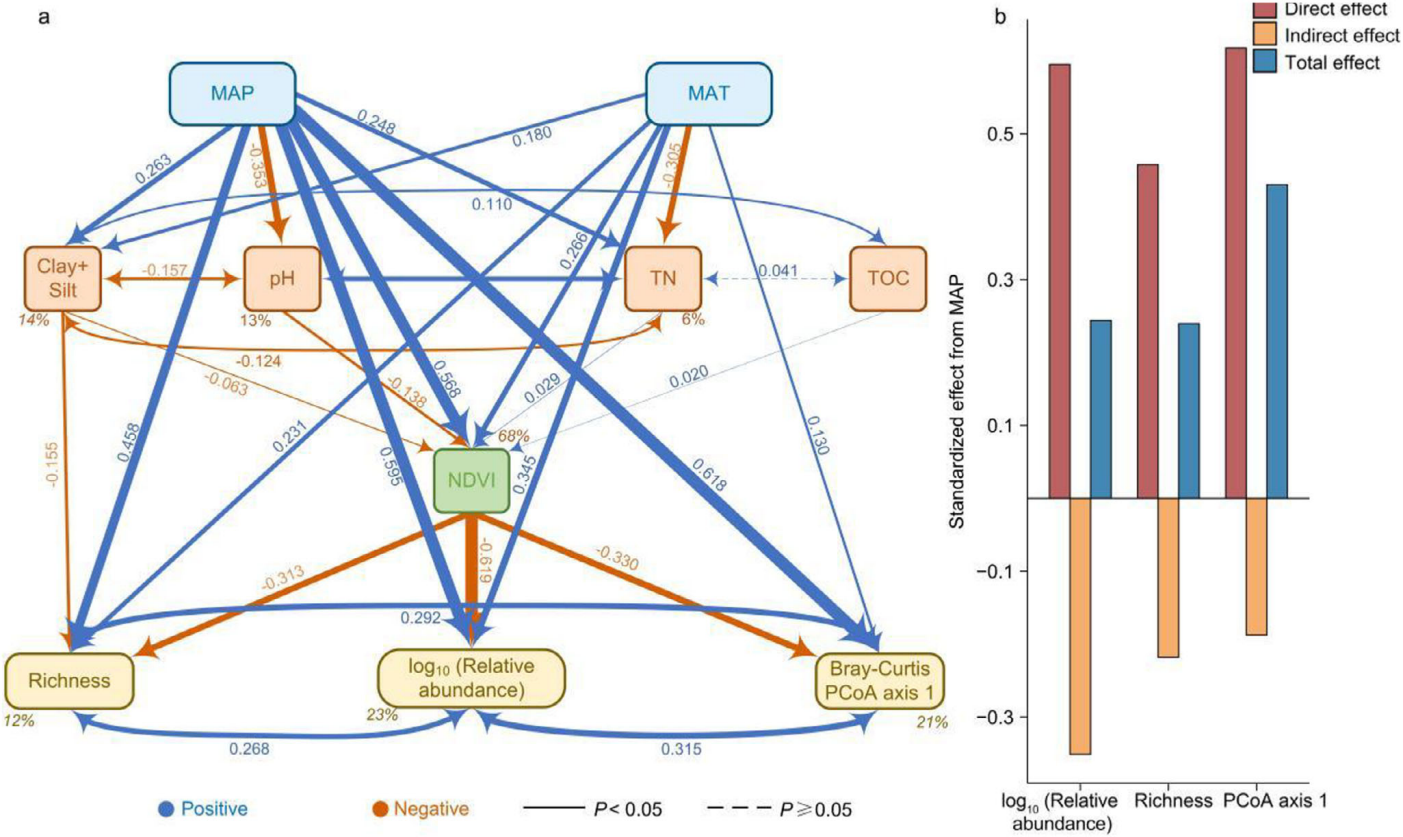
Structural equation modeling revealed the direct and indirect effects of MAP and MAT on the relative abundance, richness, and composition of potential N‐fixing microbial communities in soil (a), and the calculation of the direct and indirect effects of MAP (b). In (a), the final model fit the data well: Fisher's *C* = 30.871, df = 26, *p* = 0.233, *n* = 1253. Numbers at solid arrows (*p* < 0.05) are standardized path coefficients (equivalent to correlation coefficients), and the width of the arrows indicates the strength of the relationships. The dashed arrows indicate nonsignificant relationships (*p* > 0.05). Percentages close to variables indicate the variance explained by the model (*R* ^2^). The double‐headed arrow represents the correlation between exogenous variables. Parameter estimates from the structural equation model are shown in Table S6.

### 2.3 Low and High MAP Shaped N‐Fixing Microbiome via Deterministic and Stochastic Processes, Respectively

Within every ecosystem containing at least three replicate samples, we applied permutational multivariate analysis of dispersion (PERMDISP) to assess whether the observed N‐fixing microbial communities showed compositional similarity that differed from the theoretical similarity of stochastic communities simulated under a null model [27, 28, 29]. The observed similarity was significantly different from the theoretical similarity in 141 of 205 ecosystems (Table S7). This implied that a larger proportion of N‐fixing microbial communities were primarily driven by deterministic ecological processes, whereas a small proportion was driven by stochastic processes. Furthermore, the observed similarity was smaller than the theoretical similarity in most cases (Table S7), indicating that interspecific competition, rather than ecological filtering, was the primary deterministic process [27]. We further used the OTUs of all ecosystems as the OTU pool in the null model and calculated the standardized effect size (SES) for each ecosystem with at least three replicate samples. The SES values displayed a significant negative linear correlation with the MAP (*p* < 0.001; Figure 4a). This suggested that low MAP shaped the N‐fixing microbiome by elevating the relative role of deterministic processes (interspecific competition), while high MAP acted on it by raising the role of stochastic processes [29, 30].

**FIGURE 4:**
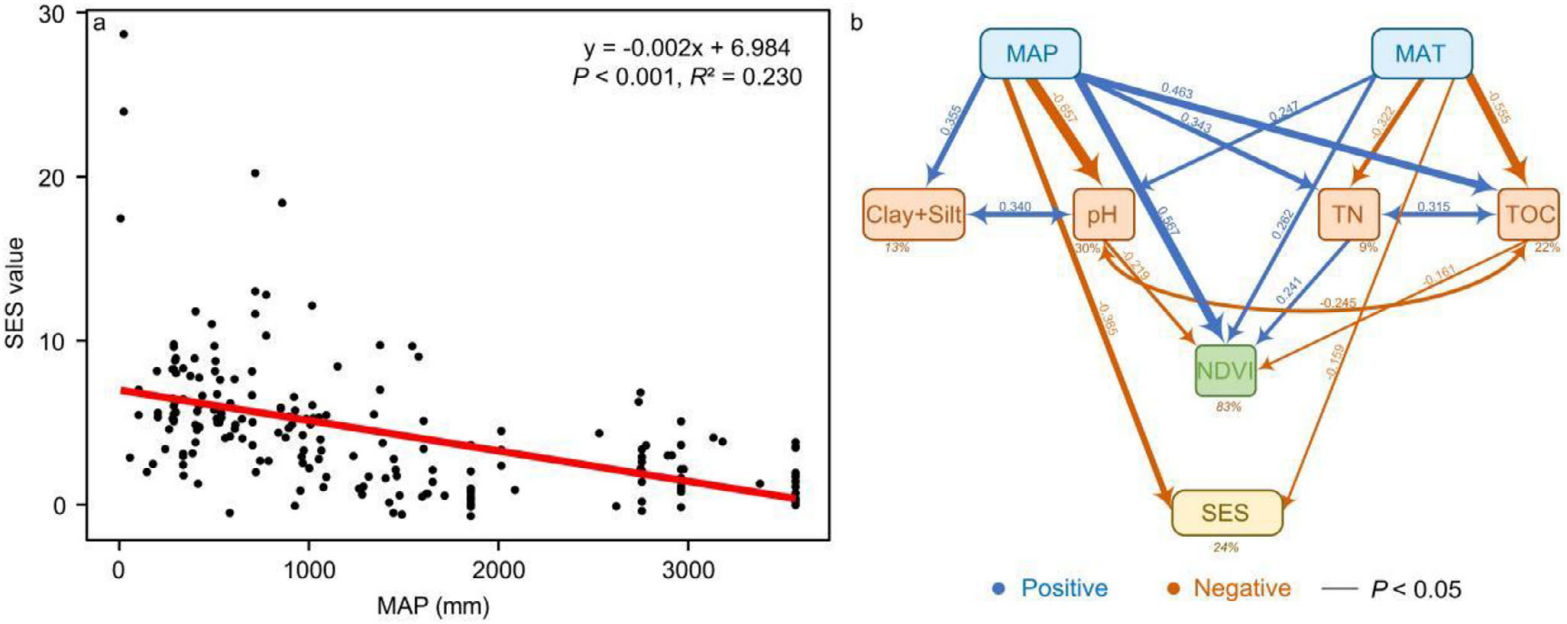
Linear correlation between the SES value of potential N‐fixing microbial community and MAP (a); structural equation modeling revealed the direct and indirect effects of MAP and MAT on the SES value (b). In (b), the final model fit the data well: Fisher's *C* = 20.572, df = 20, *p* = 0.423, *n* = 204. Numbers at solid arrows (*p* < 0.05) are standardized path coefficients (equivalent to correlation coefficients), and the width of the arrows indicates the strength of the relationships. Percentages close to variables indicate the variance explained by the model (*R* ^2^). Parameter estimates from the structural equation model are shown in Table S9.

SEM was also used to investigate the relative importance of direct and indirect routes through which the climatic factors impacted the SES of the potential N‐fixing microbial community (Table S8). The final SEM adequately fitted the data describing the effects of MAP and MAT on the SES (Fisher's *C* = 20.572, df = 20, *p* = 0.423, *n* = 204; Figure 4b and Table S9). The model explained 24% of the variation in the SES. MAP was found to have only a direct effect on the SES, and the standardized path coefficient was –0.385. It did not have an indirect effect on the SES through mediating soil and plant variables. Taken together, MAP directly rather than indirectly influenced the relative contribution of deterministic and stochastic processes to drive the assembly of the potential N‐fixing microbial community, thereby directly impacting their relative abundance, richness, and composition.

### 2.4 Low MAP Selected Larger‐Genome N‐Fixers, Whereas High MAP Favored Smaller‐Genome Species

The metagenomic sequences were also assembled into genomes, obtaining 36 bacterial genomes containing the *nifH* gene (at least medium quality; Table S10). These genomes belonged to 10 different phyla. Of these, 12 genomes belonged to the phylum Proteobacteria, renamed as Pseudomonadota; this was consistent with the taxonomic analysis of the *nifH* gene showing that this phylum had the highest relative abundance (Figure S3). Across these phyla, the genome size had a significant negative correlation with the MAP of the corresponding ecosystem (*p* < 0.05; Figure 5a). Within these genomes, the richness of the genes responsible for both carbohydrate metabolism and nucleotide metabolism displayed significant negative correlations with the MAP (*p* < 0.05; Figure 5b,c). These results indicated that low MAP selected the larger‐genome species with better functions of carbohydrate and nucleotide metabolism, whereas high MAP favored the smaller‐genome species with weaker functions. Moreover, eight genomes belonged to the same order Polyangiales of the class Polyangia of the phylum Myxococcota (Table S10). Within this order, the genome size displayed a significant negative correlation with MAP (*p* < 0.05; Figure 5d), whereas the richness of genes responsible for nucleotide metabolism showed a significant negative correlation with MAP (*p* < 0.05; Figure 5e). These results implied that low MAP selected the larger‐genome species with better nucleotide metabolism, whereas high MAP favored the smaller‐genome species with weaker nucleotide metabolism. Low MAP shaped the N‐fixing microbiome by raising the relative role of deterministic interspecific competition processes (shown previously), and microbial taxa possessing larger genomes typically exhibited greater competitive ability [23, 25]. Thus, our results demonstrated that low MAP selected the species with larger genomes through interspecific competition. Conversely, high MAP shaped the N‐fixing microbiome by elevating the relative role of stochastic processes (shown previously), and microbial taxa carrying smaller genomes typically displayed faster growth rates [24, 25]. Hence, our results demonstrated that high MAP favored the species with smaller genomes through the random birth processes. Actually, the random birth processes might result in speciation on the large temporal scale.

**FIGURE 5:**
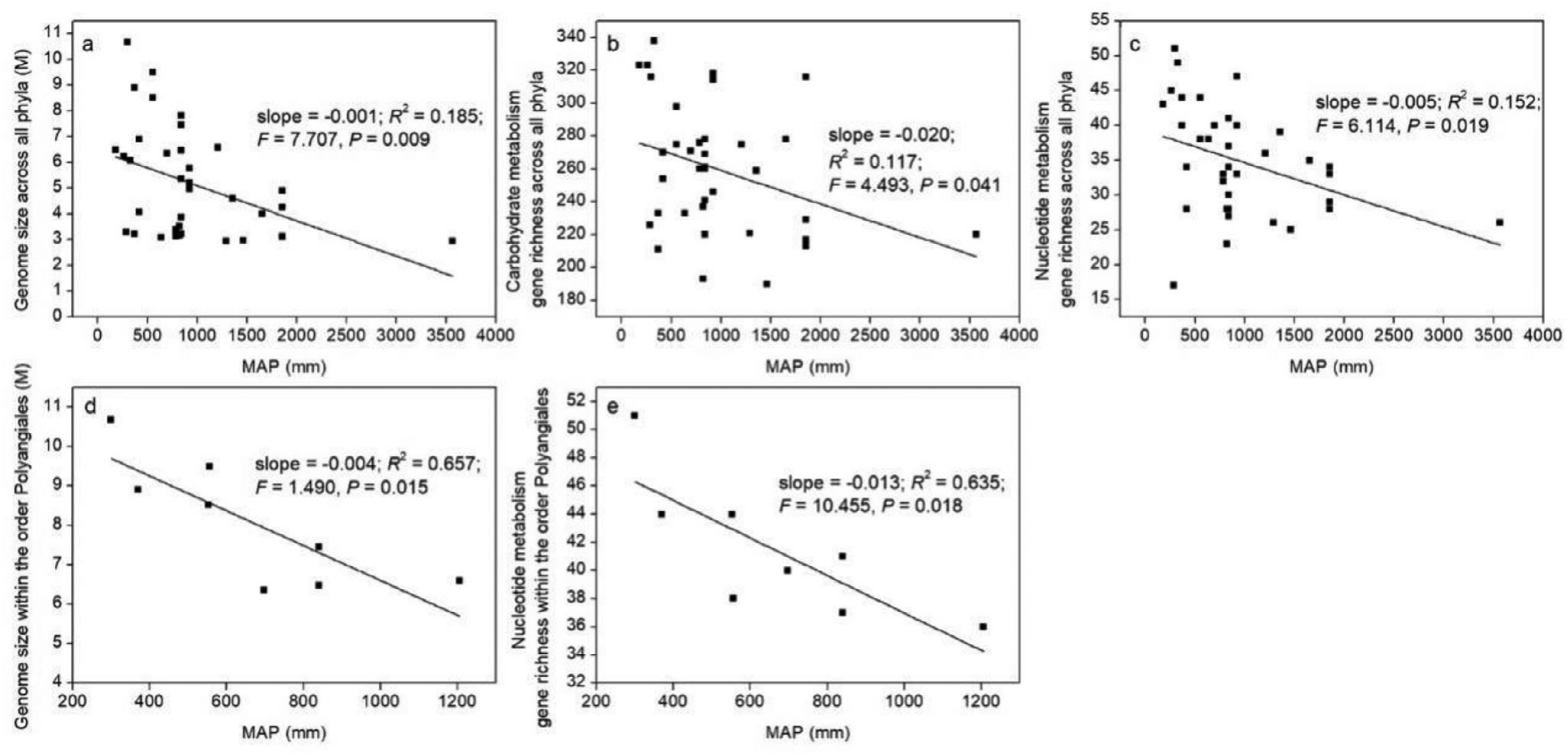
Linear correlation between MAP and the genome size or the gene richness of the carbohydrate/nucleotide metabolism function of potential N‐fixing microbial species across all phyla (a–c) or within the order Polyangiales (d,e).

### 2.5 Precipitation Increase had a Larger Regulatory Effect on the Stochastic Birth/Death Processes Compared With Other Factors in a Multifactorial Experiment

In the global survey, low MAP was found to be associated with the increased relative contribution of deterministic processes and high MAP was coupled with the elevated relative contribution of stochastic processes (Figure 4a); however, MAP per se may be closely associated with many other factors. Put differently, manipulative experiments are likely more informative than cross‐ecosystem surveys for uncovering the mechanisms behind how precipitation shifts affect the N‐fixing microbiome. Therefore, we conducted a multifactorial experiment in a semiarid grassland ecosystem in Northern China to further examine whether precipitation increase had a larger regulatory effect on the stochastic processes compared with other factors in driving the assembly of the potential N‐fixing microbial community (see the experimental design in Data S1). Briefly, the experiment comprised 13 treatments, including mowing (M), nitrogen addition (N), phosphorus addition (P), watering (W), warming (T), and some of their combination (M+N, M+P, M+W, N+P, N+W, W+T, M+N+P, and M+N+W), which simulated most of the anthropogenic environmental changes occurring in this ecosystem. The compositional variations in the potential N‐fixing microbial community between each treatment and control were separated into deterministic and stochastic components by a direct‐separation method (Data S2; Tables S3 and S11). Both the deterministic and stochastic components were negative under most environmental changes, meaning that they restrained both the directional and the non‐directional changes in the community composition (Table S12). Actually, the difference in the SES value between each treatment and control (obtained using the null model) displayed a significant positive linear correlation with the absolute value of the deterministic component (obtained using the direct‐separation method) (*p* < 0.05; Data S2; Figure S9), successfully relating the results of different methods and demonstrating their reliability.

Each environmental factor exerts a specific influence on soil microbial communities because of its particular characteristics. For example, climate warming and an increase in precipitation may impact microbial communities by increasing soil temperature and water content, respectively. Different environmental changes may also vary in intensity, which can impact the deterministic component of the variation in community composition. To separate the role of the inherent characteristic from the magnitude of the 13 environmental factors, we normalized the stochastic component of the N‐fixing community (Figure 6a and Table S12) using the formula: (the stochastic component) / (the absolute value of the deterministic component). Accordingly, the change in their community size was standardized (Figure 6b) using the formula: (the percentage change in community size relative to the control) / (the absolute value of the deterministic component) [24, 31]. A positive association was observed between the standardized stochastic component and the standardized change in community size (slope = 0.038, *F* = 34.724, *p* < 0.001, R^2^ = 0.759; Figure 6c). This suggested that the environmental perturbations altered the capacity of soil microhabitats to sustain greater or fewer microorganisms (larger or smaller community size), which in turn boosted stochastic birth or death events and enhanced the relative role of stochastic processes in shaping microbial community assembly.

**FIGURE 6:**
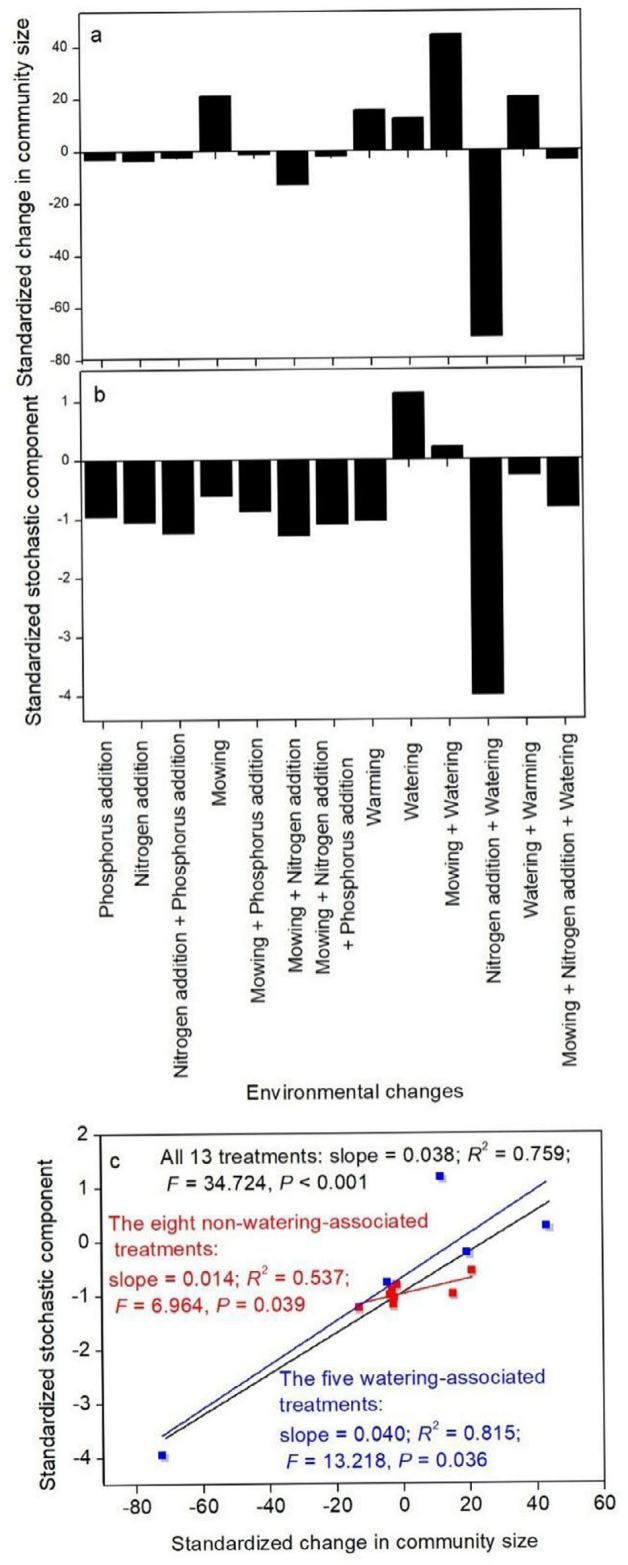
Standardized changes in community size (a) and the standardized stochastic component (b) of the potential N‐fixing microbial community under 13 anthropogenic environmental changes, and the linear relationship between them (c).

Moreover, the slope obtained for the five precipitation‐related environmental changes exceeded that of the remaining eight environmental changes substantially (permutation test: *F* = 506.5, *p* < 0.001; Figure 6c), indicating a larger regulatory effect of precipitation on the stochastic processes (and also community size) compared with other factors. Specifically, of the five precipitation‐linked environmental changes, three exhibited comparatively high values for both the standardized community‐size change and the standardized stochastic component (W, W+T, and M+W; Figure 6a,b), suggesting that they exerted a comparatively strong facilitative influence on community size and stochastic birth dynamics relative to the remaining factors. In contrast, the combination of N addition and watering (N+W; Figure 6a,b) had the smallest values of the standardized change in community size and standardized stochastic component. The advantage of N‐fixing microorganisms is their N‐fixing ability, and N addition can alleviate this advantage. Watering accelerated the diffusion of added N across the heterogeneous soil environment. Therefore, the N+W treatment had the largest reducing effect on the advantage of the N‐fixing microorganisms, thus accelerating their random death process. Despite the combination of N addition and watering in the M+N+W treatment, mowing removed aboveground plants from the experimental plot and promoted the evaporation of water from the soil. Thus, this treatment had only medium values of the standardized change in community size and standardized stochastic component (Figure 6a,b). These results demonstrated that the increase in precipitation had a larger regulatory effect on the stochastic birth/death processes compared with other factors in driving the assembly of the potential N‐fixing microbial community.

### 2.6 Estimation of the Relative Abundance and Prediction of its Change Under Various Future Climate Scenarios Globally

The eXtreme Gradient Boosting (XGBoost) model was applied to the current global climate dataset to generate a map of the current global distribution of the relative abundance of potential N‐fixing microorganisms (Figure 7a and Figure S10). This map revealed the highest relative abundance in high‐precipitation regions, such as India and Southeast Asia. Subsequently, this model served to project the relative abundance of N‐fixing soil microorganisms across four forthcoming climate scenarios [shared socioeconomic pathway (SSP) 126, sustainability; SSP245, middle of the road; SSP370, regional rivalry; and SSP585, fossil‐fueled development up to 2040] by applying 14 distinct Coupled Model Intercomparison Project 6 (CMIP6) downscaled global‐change models, thereby reducing discrepancies among the individual simulations (Table S13). The relative abundance of potential N‐fixing microorganisms in soil increased in most regions on Earth, such as the vast majority of the regions in Australia (Figure 7b–e), indicating that they could relax soil N limitation at least partly. By the year 2040, the relative abundance across all terrestrial ecosystems globally is projected to increase by 2.475%, 2.686%, 2.645%, and 3.522% under the SSP126, SSP245, SSP370, and SSP585 scenarios, respectively (Figure 7f). Also, the relative abundance will continue to increase with time. By the year 2100, it will increase by 4.653%, 11.203%, 18.830%, and 24.345% under the SSP126, SSP245, SSP370, and SSP585 scenarios, respectively (Figure 7f). However, under these future climate scenarios, the relative abundance of potential N‐fixing microorganisms is still projected to decrease in certain regions, such as Northern Africa (Figure 7b–e), indicating that additional N‐fixing microbial agents or N fertilizers may be needed in these regions to maintain soil N supply and support plant productivity.

**FIGURE 7:**
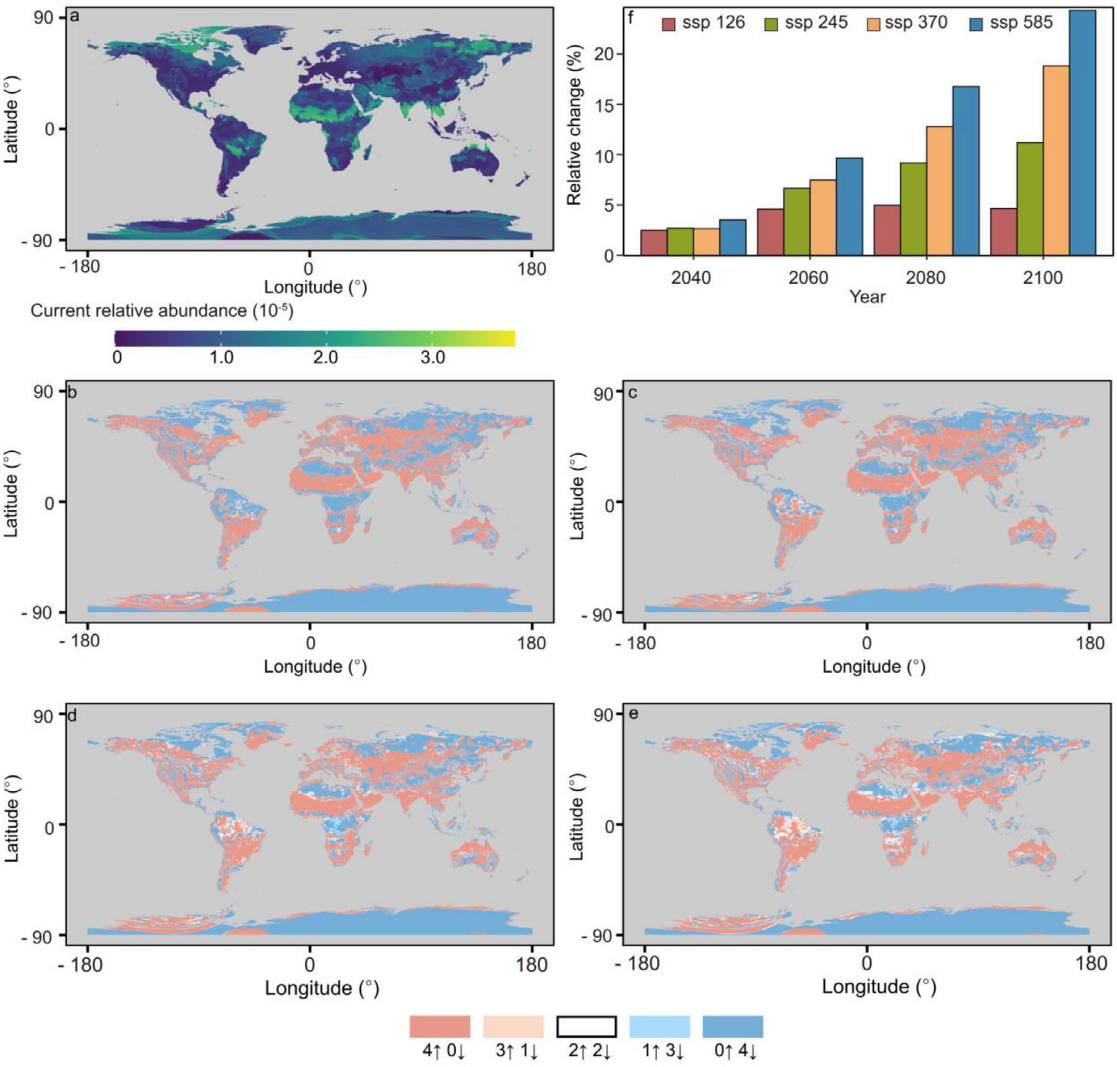
Global map of the predicted contemporary relative abundance of soil N‐fixing microbial communities (a), global maps showing the change trend of the predicted relative abundance under four future climate change scenarios for the years 2040 (b), 2060 (c), 2080 (d), and 2100 (e), and the overall global relative change in the predicted relative abundance under each scenario across these timepoints (f). The four scenarios included SSP126 (sustainability), SSP245 (middle of the road), SSP370 (regional rivalry), and SSP585 (fossil‐fueled development).

## 3 Conclusions and Limitations

Exploring the biodiversity and biogeography of soil microbial groups with important ecological functions globally is a key objective in contemporary microbial ecology [5, 18, 32, 33]. Through the compilation of worldwide soil metagenomic datasets, assembly of a complete *nifH* reference gene catalog, and alignment of metagenomic reads against this catalog, our work revealed the distinctive pattern and mechanism of the biodiversity and biogeography of potential N‐fixing soil microorganisms. Based on these results, the theoretical framework of MAP driving the biodiversity and biogeography of soil N‐fixing microbial communities globally was constructed (Figure 8).

**FIGURE 8:**
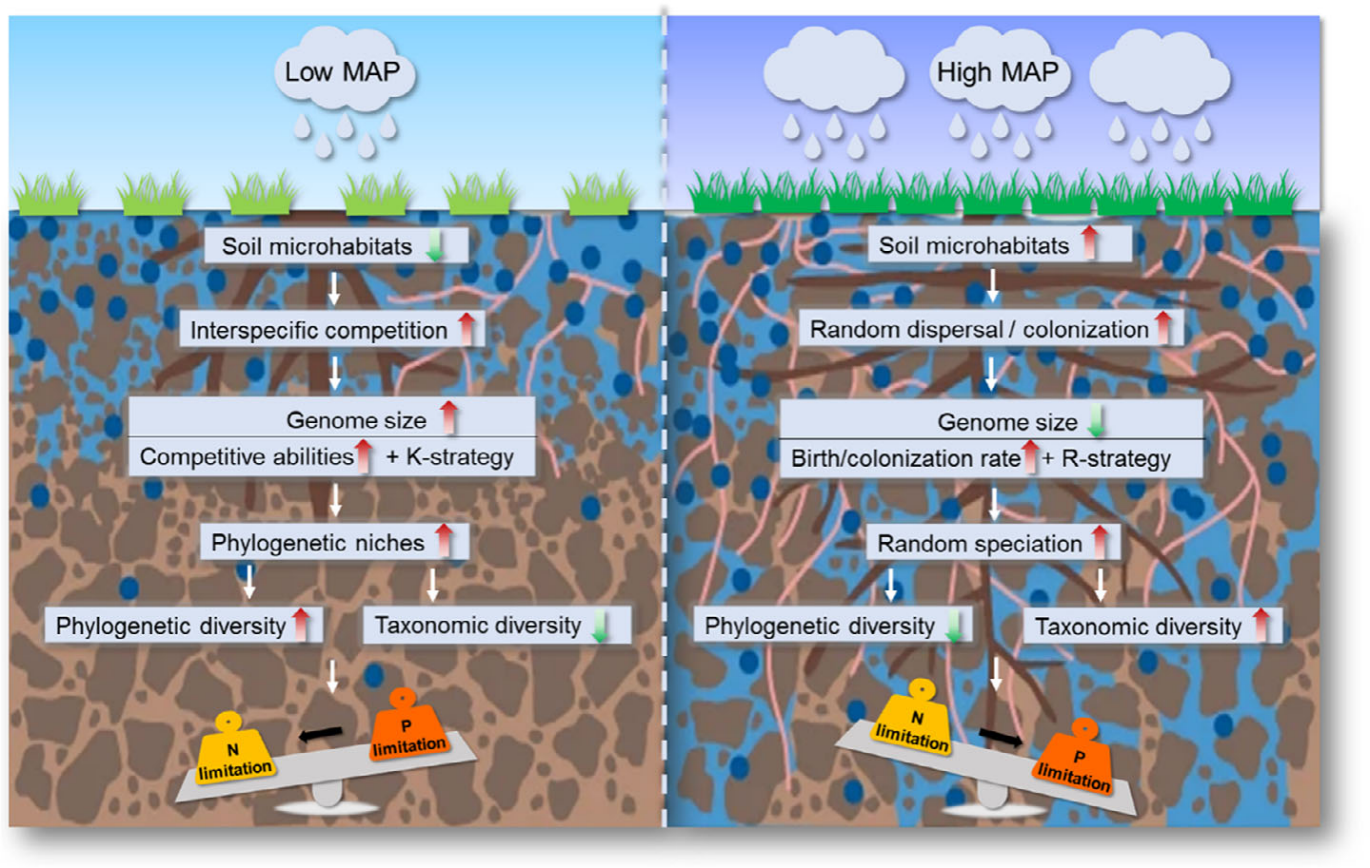
Conceptual framework illustrating the impact of MAP on the potential N‐fixing microbial community in soil.

These microorganisms can live only in soil microhabitats containing water. MAP directly determined the number of such microhabitats. Consequently, across a range of climatic, vegetation, and edaphic physicochemical variables, MAP emerged as the predominant determinant of the relative abundance, diversity, and structure of soil N‐fixing microbial communities worldwide (Figure 8). Low MAP reduced the number of soil microhabitats and intensified interspecific competition, thus selecting the larger‐genome species with stronger competitive abilities, akin to the K‐strategist [22, 25]. In contrast, high MAP increased the number of soil microhabitats and promoted the random dispersal/colonization of species, thus favoring the smaller‐genome species with higher birth/colonization rates, akin to the R‐strategist [22, 25]. The interspecific competition under low‐MAP conditions led to the exclusion of many species with similar phylogenetic niches, resulting in decreased taxonomic diversity but increased phylogenetic diversity of the community. The random birth of smaller‐genome species under high‐MAP conditions might lead to random speciation over larger temporal scales, and the new species had extremely close phylogenetic relationships with their ancestral species. Meanwhile, the random birth/speciation of smaller‐genome species might result in the random death/extinction of larger‐genome species. Thus, these processes together led to increased taxonomic diversity but decreased phylogenetic diversity (Figure 8). The decreased relative abundance and taxonomic diversity of N‐fixing microorganisms under low‐MAP conditions suggested that soil N limitation might become more severe, emphasizing the need for applying N fertilizer or N‐fixing agents to maintain ecosystem productivity. The increased relative abundance and taxonomic diversity of N‐fixing microorganisms under high‐MAP conditions indicated that soil N limitation might be relaxed, but other elements (e.g., phosphorus and trace elements) might be more limited, which requires further investigation.

Overall, this study demonstrated the distinctive pattern and mechanism of MAP driving the global biodiversity and biogeography of potential N‐fixing microorganisms in soil. However, MAP is very likely to be closely associated with many other factors (e.g., MAT) in affecting the N‐fixing microbiome in the global survey. In contrast, there are more explicit causal relationships between the treatment factors and the research subjects in the manipulative experiment. Thus, more manipulative experiments with multiple precipitation gradients should be conducted in the future to further examine the pattern and mechanism. In particular, more time‐series samples are needed to monitor the random birth/death processes. In addition, more evidence (e.g., comparative assays of bacterial strain competitiveness on agar plates) is needed to further examine the deterministic competitive mechanism under low precipitation conditions in future studies.

This study only focused on the drivers and mechanisms of N‐fixing microorganisms among different ecosystem types globally and highlighted the importance of MAP. When focusing on a single ecosystem type (e.g., tropical forests), the variation in MAP becomes narrower, and the underlying drivers and mechanisms may differ, highlighting the need for further investigation.

This study only aimed to investigate the most important driver for the global soil N‐fixing microbiome, without delving into the influence of other factors. Actually, our results did suggest that MAT was the second most important factor for soil N‐fixing microorganisms at the global scale (Figure 2 and Figure S11). Future studies should be conducted to examine the effects and mechanisms of MAT and other factors on the global soil N‐fixing microbiome. Furthermore, the potential interactive effects of all these factors on the N‐fixing microbiome should also be studied.

When MAP was identified to be the primary driver for the N‐fixing microbial community on the global scale, variables associated with soil N content (e.g., total N (TN) content and available N content) were found to be not important, which was in contrast to the intuitive hypothesis that certain variables associated with soil N content should be very important in restraining the N‐fixing microbiome. Soil TN content was not important (Figure 2), which is possibly because the majority of the TN in the soil is not easily decomposable or accessible by microorganisms. Soil available N content was still found to be not important (Figure 2 and Figure S6), which was likely due to that the available N content in these undisturbed natural ecosystems was so small as to cause a restraining effect on N‐fixing microorganisms. In other words, soil available N content will be important to restrain the N‐fixing microbiome only when it is above a certain threshold, which also needs further examination in the future.

## 4 Materials and Methods

### 4.1 Global Soil Metagenomic Sequencing Data Collection

We retrieved the metagenomic sequence datasets from the National Center for Biotechnology Information (NCBI) (www.ncbi.nlm.nih.gov) through searches with the terms “WGS” and “SOIL” through July 2021. All the downloaded datasets were manually checked. Samples from non‐soil environments, laboratory microcosmic experiments, and wetlands, or samples with incomplete information on latitude and longitude, or those with data volume less than 1G were removed. A total of 585 projects with 3211 soil metagenomic samples were eventually retained for further analysis. Of these, 1876 were natural control samples rather than samples from experimental treatments (e.g., N or water addition).

### 4.2 Soil Sample Collection and Measurement in China

A total of 30 field ecosystems, representing 9 ecosystem types in the Chinese mainland, were selected for soil sampling (Table S1). Only soils unsaturated for the majority of the year were sampled. Soil samples were collected during peak plant biomass, specifically from mid‐ to late August 2019. For each ecosystem, a square‐form quadrat with a side of about 120 m was selected, and a soil sample was taken from each of the four corners. A soil drill with a diameter of 3.5 cm was used to collect soil samples at a depth of 0–20 cm. A total of 120 soil samples were collected. Roots and stones were first excluded by passing samples through a 4‐mm sieve, after which the samples were held in an ice‐packed cooler and dispatched to our Beijing laboratory within a few days of retrieval. Subsequently, every soil sample was partitioned into three aliquots: one was stored frozen at –20°C for genomic DNA isolation; a second was maintained at 4°C for assays of dissolved organic carbon (DOC), available N (NH_4_^+^‐N and NO_3_^−^‐N), and water content; and the remaining portion was air‐dried prior to determination of pH, total carbon (TC), total nitrogen (TN), total phosphorus (TP), available phosphorus (AP), available potassium (AK), plus clay and silt fractions.

We isolated DOC by combining 12.5 g of homogenized soil subsamples with 50 mL of 0.5 m potassium sulfate (K_2_SO_4_), followed by 1 h of agitation at 120 rpm on an orbital shaker. The filtrate was analyzed using a TOC analyzer (high TOC, Elementar). Soil available N content was extracted with 1 mol L^−1^ KCl and then determined using a Foss FIAstar 5000 flow injection auto‐analyzer (Foss Tecator, Hillerød, Denmark). The soil water content was determined as the weight loss after drying for 24 h at 105°C. Soil pH was determined in aqueous suspensions prepared at a 1:2.5 (w/v) soil‐to‐water ratio using distilled water. The TC and TN contents were quantified using the potassium dichromate–vitriol oxidation method and the Kjeldahl acid‐digestion method, respectively [34]. The soil TP content was measured by the ammonium molybdate method after persulfate oxidation [34]. Soil AP was first extracted with 0.5 m NaHCO_3_ and then assessed via the ammonium molybdate–ascorbic acid blue procedure. Absorbance was subsequently read at 700 nm on a Varioskan LUX microplate reader (Thermo Scientific, USA) [35, 36]. The AK content was determined by 1 mol·L^−^^1^ ammonium acetate extraction–flame photometry. The soil clay and silt percentages were determined by the method of soil particle‐size fraction [37].

Genomic DNA was isolated from soil using the FastDNA SPIN Kit for Soil (MP Biomedicals, USA), then sheared to ∼150 bp with a Covaris M220 ultrasonicator (Gene Company Limited, China) ahead of metagenomic library construction. We employed the NEXTFLEX Rapid DNA‐Seq Kit (Bioo Scientific, TX, USA) to build the paired‐end library. Metagenomic sequencing was performed with the DNBSeq G400 platform by Beijing Genomics Institute Genomics (Shenzhen, China).

### 4.3 Construction of a Full‐Length *nifH* Reference Gene Set

Raw reads from every downloaded and self‐tested metagenome were passed through FASTP (v0.23.1) quality trimming with the settings ‘‐q 20 ‐W 50 ‐cut_tail ‐M 20 ‐l 50 ‐n 1' [38]. Subsequently, the filtered reads from every sample were de novo assembled into contigs via MEGAHIT, applying the settings ’–k‐min 47 –k‐max 97 –k‐step 10 –min‐contig‐len 300' [39]. Open reading frames (ORFs) within the assembled contigs were identified with MetaGeneMark (https://github.com/gatech‐genemark/MetaGeneMark). The full‐length gene sequences of ORFs were obtained by screening for sequences ending with termination codons (TAG, TAA, and TGA). The nonredundant full‐length gene sequences were generated by deduplicating the redundant full‐length gene sequences with 100% similarity using SeqKit—rmdup. Gene functions were annotated by aligning sequences against the NCycDB database with DIAMOND (version 2.0.15), adopting an e‐value threshold of 1e‐5 and an identity threshold of 50% [40, 41]. Finally, 6607 nonredundant representative full‐length sequences of the *nifH* gene (Table S14) were obtained. Taxonomic assignment of these sequences was subsequently carried out by matching them against the NR database through DIAMOND (version 2.0.15) under an e‐value threshold of 1e‐5.

### 4.4 Mapping of Reads and Calculation of *nifH* Gene Richness

For each of the 1876 downloaded control samples and 120 self‐collected samples, the reads were mapped to *nifH* nonredundant representative full‐length sequences at 100% similarity using BWA (https://github.com/lh3/bwa). The abundance of each nonredundant representative full‐length *nifH* sequence was calculated as reads per kilobase (RPK), using the number of mapped reads and gene length: $RPK=10^{3\;}\times \;\frac{total\;reads\;mapped\;to\;the\;gene}{gene\;length\;in\;bp}$. The nonredundant representative full‐length sequences of the *nifH* gene were clustered with 97% similarity into OTUs using QIIME2‐vsearch [42]. Samples with a sum of *nifH* abundance less than 40 were removed, finally retaining 1257 samples. For each sample, 40 reads were randomly drawn for calculating OTU richness and for all subsequent analyses (Table S2). Similarly, the samples with a sum of *nifH* abundance less than 200 were also removed, retaining 576 control samples. 200 reads were randomly drawn for calculating OTU richness and for some of the following analyses.

### 4.5 Construction of the Phylogenetic Tree

The full‐length *nifH* reference gene sequence was aligned using MUSCLE v5.1 [43]. Subsequently, the multiple sequence alignment was fed into FastTree v2.1.11 (default parameters) [44], and a maximum likelihood (ML) tree was constructed. Based on the phylogenetic tree, the phylogenetic diversity (PD) of each sample was calculated using the “picante” R package [45]. The unweighted UniFrac distance between any two samples was calculated using the “GUniFrac” R package.

### 4.6 Metagenome‐Assembled Genome Reconstruction and Characterization

Metagenome‐assembled genomes (MAGs) were reconstructed by binning contigs using MetaBAT2 (v2.15) [46]. The genome quality was evaluated using CheckM v1.2.2, retaining only MAGs meeting the following thresholds: >50% completeness and <10% contamination [47]. ORF prediction was performed on quality‐filtered MAGs using Prodigal (v2.6.3) [48] in the metagenomic mode (‐p meta). The functional annotation was conducted with eggNOG‐mapper (v2.1.12) [49] using default parameters to identify MAGs encoding the *nifH* gene. For MAGs harboring *nifH*, ribosomal RNA genes were annotated using Barrnap (v0.9) (https://github.com/tseemann/barrnap) with default parameters. The taxonomic annotation was performed using GTDB‐Tk (v2.3.2) with the “wf_classify” workflow [50] against the Genome Taxonomy Database (release 214).

### 4.7 Compilation of Climatic, Vegetation, and Soil Property Information

The 1257 samples (each containing at least 40 *nifH* reads) were from 318 ecosystems. For each sample, its ecosystem type was identified based on its latitude and longitude coordinates by combining the Köppen climate classification (https://hanschen.org/koppen/) and the FAO (Food and Agriculture Organization of the United Nations) land cover and land use data (https://data.apps.fao.org/catalog/dataset/7f4f5ce4‐9a3e‐406f‐bae6‐efe63867329a). Measurements of soil characteristics—namely pH, TOC, clay, and silt contents—were obtained from the Harmonized World Soil Database v.1.2. The data on TN content were downloaded from DAAC (https://daac.ornl.gov/cgi‐bin/dsviewer.pl?ds_id = 569). The normalized difference vegetation index (NDVI) records were retrieved from NASA's EarthData portal (https://lpdaac.usgs.gov/products/mod13c2v006/) as a proxy for vegetation net primary productivity. Climate variables—potential evapotranspiration (PET), MAT, and MAP, among them—for every ecosystem were obtained from the WorldClim database (www.worldclim.org) on the basis of the latitude and longitude of each site. Projected future climate variables (for 2040, 2060, 2080, and 2100) were drawn from the downscaled CMIP6 model outputs (https://esgf‐node.llnl.gov/projects/cmip6/). The monthly values of minimum temperature, maximum temperature, and precipitation were processed for four SSPs: 126, 245, 370, and 585. Detailed explanations of the SSP scenarios can be found at https://www.carbonbrief.org/explainer‐how‐shared‐socioeconomic‐pathways‐explore‐future‐climate‐change. The climate projections under each SSP scenario were generated using 14 downscaled global climate models from CMIP6 (Table S13).

### 4.8 Statistical Analyses

Data normality distribution was tested using the Kolmogorov–Smirnov test. The Kruskal‐Wallis non‐parametric test (applicable to non‐normally distributed data) was used to determine statistically significant differences in the relative abundance and richness (or PD) of the potential N‐fixing microorganisms among different ecosystem types. PCoA was conducted to exhibit the compositional variations in N‐fixing microbial communities with the “vegdist” function of the “vegan” package [51]. We employed PERMANOVA to detect variations in community composition among ecosystem types, using either a Bray–Curtis dissimilarity matrix or an unweighted UniFrac distance matrix.

We fitted linear mixed‐effects models using the “lmer” function from the “lme4” R package to determine the primary environmental factors shaping the richness, relative abundance, and community composition (PCoA axis 1) of these potential N‐fixing microorganisms [52]. The variables related to climate (AI, MAT, and MAP), soil properties (pH and TOC, TN, clay, and silt contents), and vegetation (NDVI) were tested for collinearity, and the variables with VIF values <10 were set as fixed factors in models, and the sampling site was set as a random factor. To dissect the contributions of these predictors, we applied the “glmm.hp” package to decompose their variance [53]. We carried out distance‐based redundancy analysis (dbRDA) to assess how strongly each of these environmental factors contributed to the N‐fixing microbial community structure, using a Bray–Curtis dissimilarity distance matrix [51].

Using the R package “piecewiseSEM”, we applied SEM to explore linkages between climatic, edaphic, and vegetation factors (MAT, MAP, NDVI, TN, TOC, pH, clay, and silt) and the richness, relative abundance (log10‐transformed), and community structure (PCoA axis 1) of soil N‐fixing microbes [54]. The collinearities among variables were tested first, and the variables with a variance inflation factor (VIF) value <10 were selected. The prior model was constructed based on both prior knowledge and the results of previous mixed linear models (Table S5). Mixed‐effect models were applied in the SEM using the “lme” function from the “nlme” package [55], as the “site” was set as the random effect. The missing paths in the initial model were detected using Shipley's test of directed separation (d‐separation test). From the identified missing paths, we selectively added those that were both statistically significant and ecologically plausible to the model. Subsequently, we iteratively removed the least significant paths. On each iteration, the SEM was refitted and one path removed, and the Akaike information criterion (AIC) and Fisher's C p‐value were evaluated. ​The iterative removal was terminated when the final model was selected as the simplest structure and fitted the conditional independence claims at 95% confidence (Fisher's *p*‐value **>** 0.05).

The difference between the compositional similarity of observed communities and the theoretical compositional similarity of stochastic communities from the null model (Data S3) was tested with the PERMDISP for each sampling location with at least three replicate samples [27, 28, 29]. The nonsignificant difference between the observed community and the random community indicated the dominance of stochastic processes in driving community assembly [27, 29]. The significant difference indicated the dominance of deterministic processes. Specifically, the larger similarity in the observed community than in the random community indicated environmental filtering, whereas the larger similarity in the random community than in the observed community indicated interspecific competition (Data S3). Furthermore, on the global scale, all OTUs from all locations were used as the OTU pool in the null model, and the SES was calculated for each location with at least three replicate samples [27, 28, 29]: SES = (J_obs—_ $\bar{J}$_exp_)/σ_exp_). The linear regression was adopted to analyze the effect of MAP on the SES values. SEM was also conducted to analyze the relationships among climatic, environmental, and vegetational variables and the SES using the R package “piecewiseSEM” [54]. The prior model is shown in Table S8.

Nineteen bioclimatic variables were extracted from the WorldClim database for each sampling location, representing average climate conditions from 1970 to 2000. These included 11 temperature‐related and 8 precipitation‐related variables. Future climate projections were obtained from downscaled CMIP6 (Coupled Model Intercomparison Project Phase 6; https://esgf‐node.llnl.gov/projects/cmip6/) data for four time periods: 2021–2040, 2041–2060, 2061–2080, and 2081–2100 (Table S13). We extracted bioclimatic predictors for the four Shared Socio‐economic Pathways—namely SSP126 (sustainability), SSP245 (middle of the road), SSP370 (regional rivalry), and SSP585 (fossil‐fueled development). We used XGBoost (through the “xgboost” package) to forecast the worldwide distribution of relative abundance of the N‐fixing microbiome [56]. Before making predictions, we ran leave‐one‐out cross‐validation (LOOCV) to assess the model's predictive performance and detect possible overfitting. The LOOCV results showed a strong performance with an *R* ^2^ of 0.677 (*p* < 0.001, Figure S10). Using the XGBoost model trained on current climate data, we further projected global distribution patterns of relative abundance under different future climate scenarios. Future projections were based on climate data from 14 downscaled global climate models (GCMs) in the CMIP6 framework, with relative changes averaged across these GCMs. To quantify the projected changes under climate scenarios, we employed a consistency metric across four climate scenarios for each time period. For every grid cell, the predicted value in each period was benchmarked against the present‐day baseline. We subsequently tallied how many scenarios showed projected values exceeding or falling short of the baseline.

Further, a multifactorial experiment was conducted in a semiarid grassland ecosystem in Northern China to examine whether the precipitation increase had a larger regulatory effect on the stochastic processes compared with other factors in driving the assembly of the N‐fixing microbial community (see the detailed experimental design and data analyses in Data S1 and Data S2).

## Author Contributions

X.Z. conceived the project and designed the experiments. B.H., S.P., A.L., and H.W. performed the experiments. B.H., Z.H., and S.Z. analyzed the data. B.H., S.P., and X.Z. wrote the manuscript. All authors contributed to the discussion and revision of the manuscript.

## Funding

This study was supported by the National Natural Science Foundation of China (U21A20188), the Double Thousand Plan of Jiangxi Province (jxsq2023102216), and the Top‐Notch Young Talents Program (to Ximei Zhang) of China.

## Ethics Statement

The authors have nothing to report.

## Consent

The authors have nothing to report.

## Conflicts of Interest

The authors declare no conflicts of interest.

## Supporting information




**Supporting File 1**: advs77215‐sup‐0001‐S1‐S3.docx.

**Supporting File 2**: advs77215‐sup‐0002‐S1‐S11.docx.

**Supporting File 3**: advs77215‐sup‐0003‐S1.xlsx.

**Supporting File 4**: advs77215‐sup‐0004‐S2.xlsx.

**Supporting File 5**: advs77215‐sup‐0005‐S3.xlsx.

**Supporting File 6**: advs77215‐sup‐0006‐S4.xlsx.

**Supporting File 7**: advs77215‐sup‐0007‐S5.xlsx.

**Supporting File 8**: advs77215‐sup‐0008‐S6.xlsx.

**Supporting File 9**: advs77215‐sup‐0009‐S7.xlsx.

**Supporting File 10**: advs77215‐sup‐00010‐S8.xlsx.

**Supporting File 11**: advs77215‐sup‐00011‐S9.xlsx.

**Supporting File 12**: advs77215‐sup‐00012‐S10.xlsx.

**Supporting File 13**: advs77215‐sup‐00013‐S11.xlsx.

**Supporting File 14**: advs77215‐sup‐00014‐S12.xlsx.

**Supporting File 15**: advs77215‐sup‐00015‐S13.xlsx.

**Supporting File 16**: advs77215‐sup‐00016‐S14.xlsx.

## Data Availability

The metagenome sequencing data of the survey in this study were deposited in the National Genomics Data Center (NGDC, https://ngdc.cncb.ac.cn/) under the project accession number PRJNA1287550. The metagenome sequencing data of the multifactorial experiment were deposited in NGDC under the project accession number PRJNA1290526.
