# Supplementary material for: Rainfall Shapes the Diversity of Soil Nitrogen‐Fixing Microorganisms Worldwide
Source: Adv Sci (Weinh). 2026 Aug 17:e77215. Online ahead of print. doi: 10.1002/advs.77215 (PMC13479853; doi:10.1002/advs.77215)
Supplement: Supplementary file 1 [file ADVS-9999-e77215-s013.docx]

**Data S1. The multifactorial experiment.**

This experiment was conducted in a semiarid grassland ecosystem in Inner Mongolia, China (116°14’E, 43°13’N). A total of 13 treatments were employed in a partially nested design: including mowing (M), nitrogen addition (N), phosphorus addition (P), watering (W), warming (T), and some of their combination (M+N, M+P, M+W, N+P, N+W, W+T, M+N+P, and M+N+W), which simulated most of anthropogenic environmental changes occurring in this ecosystem. The experiment contained three parts, and we provide a brief description here. In the first experimental part, the influences of mowing (M), adding nitrogen (N), adding phosphorus (P), and their combinations were investigated. M was as the primary factor and nutrient (N and P) addition were as the secondary factor. Four primary plots were randomly assigned to the M treatment and the other four were controls. M treatment was carried out by weeding out aboveground plants on 20 August every year. Each of the four secondary plots was randomly assigned to adding N, adding P, adding N and P, and control treatments. N treatment was added urea in 2015 and NH_4_NO_3_ from 2016 to 2020 with a rate of 10 g N m^-2^ yr^-1^, which was estimated as N deposition rates in this region. P treatment was added in the form of calcium superphosphate at a rate of 5 g PO_4_^3-^m^-2^ yr^-1^. N and P addition was carried out on a rainy day in the July every year. There were eight treatments in total and four replicates for each treatment.

In the second part, the influences of mowing (M), adding nitrogen (N), watering (W), and their combinations were investigated, with eight treatments in total and four replicates for each treatment. Within each of the secondary plots which were carried out with N addition or control, two 10m × 15m third-level plots were established, with one third-level plot being un-watered and the other being watered during summer (July and August) in 2015-2020. In the watering treatment, 15 millimeters of water was sprinkled weekly, thus about 120 mm water was added every year. The second part partially overlapped the first experiment.

In the third part, the influences of warming (T), watering (W), and their combinations were investigated, with a total of four treatments and six replicates for each treatment. First, we randomly selected three control (without M) primary plots, each of which contained a control (neither add N or P) secondary plot. Within each of these three control secondary plots, there were two third-level plots, with one being watered and the other being un-watered. Within each of the six third-level plots, four 3m × 4m fourth-level plots were set up, with two being randomly assigned to T treatment and the other two being controls. The warmed plots were heated by using 165cm × 15cm MSR-2420 infrared radiators (Kalglo Electronics, Bethlehem, PA, USA) suspended 2.5m above the ground from 28 April 2015, which increased mean soil temperature 1.80°C higher within the warmed plots than the control plots. The third part partially overlapped the second part. These treatments were continuously applied every year since 2015, and we sampled in 2020.

Surface soil samples were taken on late August 2020. Four soil cores (10 cm depth, 3.5 cm diameter) were collected at random locations in each plot, and thoroughly mixed. After passing through a 1mm sieve to removing visible roots and stones, then a small part of soil samples was frozen for DNA extraction. For each soil sample, we extracted DNA from 0.5g of mixed soil according to the manufacturer’s instructions of the Fast DNA SPIN kit for soil (Qbiogene, Carlsbad, CA, USA). The DNA solution was stored at -20°C for microbial analysis. Metagenome sequencing and bioinformatics analyses were performed similarly as the 120 China samples as described in the main text, obtaining the compositional data of the potential N-fixing microbial communities (Table S3). Meanwhile, soil bacterial 16S rRNA gene copy number was quantified using the real-time polymerase chain reaction. Accordingly, the absolute abundance (community size) of the *nifH* gene was estimated as the product of soil bacterial 16S rRNA gene copy number and the relative abundance of the *nifH* gene (from the metagenome data).

**Data S2. Calculating the deterministic and stochastic components of the community compositional variations with the direct-separation method.**

Briefly, because the differences between the control and treatment communities should be primarily caused by deterministic processes induced by the treatment factor, the *deterministic component* was quantified as: [(mean compositional variation between treatment and control) - (mean compositional variation within control)] (Zhang *et al.*, 2011; Zhang *et al.*, 2016). The effects of soil physicochemical indices and plant community indices on within-treatment compositional variation of microbial community were tested, and the effects were insignificant for almost all treatments (*P* > 0.05; Table S11), which indicated that the deterministic effects were equal among replicate plots within a treatment, so the compositional variations between plots within a treatment should be caused primarily by stochastic processes. Thus, the *stochastic component* was estimated as: [(mean compositional variation within treatment) - (mean compositional variation within control)].

To test whether the deterministic component was statistically significant for each treatment, we calculated the mean compositional variation between each treatment plot and all control plots. Similarly, we also calculated the mean compositional variation between each control plot and all other control plots. For each treatment, independent-sample *t*-test was used to analyze whether there was significant difference between this kind of mean compositional variations of the treatment and those of the control. To test whether the stochastic component was statistically significant for each treatment, we tested whether there was significant difference between the within-treatment compositional variation and the within-control compositional variation, using the nonparametric permutation analysis method of permutation dispersion.

To examine the results from the direct-separation method, we also analyzed the data of the 13 treatments with the null model method, similar to the analysis for the global survey data as described in the main text. The direct-separation method compared the treatment communities with the control communities and thus separated the deterministic and stochastic components of the treatment effect (Zhang *et al.*, 2011; Zhang *et al.*, 2016). In contrast, the null model method compared the observed communities to the stochastically assembled communities, and thus assessed the deterministic effect caused by both current experimental treatment and past historical factors (such as the environmental changes occurred before the experimental treatment). The difference in the SES value between each treatment and control (from the null model method) was found to show a significant positive linear relationship with the absolute of the deterministic component (from the direct-separation method) (*P* < 0.05; Fig. S8), successfully linking the results of different methods and demonstrating their reliability.

**Data S3. Analysis of the relative contribution of deterministic vs. stochastic processes with a null model method.**

We estimated the relative contribution of deterministic vs. stochastic processes in driving the assembly of N-fixing microbial community with a null model method. β diversity, which represents the compositional variation between communities, is often used to infer mechanisms of community assembly, such as the relative importance of deterministic vs. stochastic processes. However, differences in β-diversity indexes may be caused by differences in ecological processes, as well as α and γ diversity. To account for the effect of the other two diversity components, Chase *et al.* (2010) developed a null model method that compares the observed β diversity to the theoretical β diversity from stochastically assembled communities. Here we analyzed the community data of each site following the methods of Chase (2010) and Zhou *et al.* (2014). First, for any given pair of plots within a site, we calculated the observed species richness (e.g., α1 and α2 for plot 1 and 2, respectively) and the number of shared species (SSobs). Second, the total number of species detected in the “species pool” (γ diversity) from all plots of a site and the proportion of the plots occupied by each species were measured. Third, we calculated the distribution of the expected shared species from the null model (SSexp) by randomly drawing α1 and α2 species from the species pool, and the probability of a species drawn was proportional to its among-plot occupancy. The SSexp and the expected Jaccard’s similarity (Jexp) were obtained for each draw, and the average Jaccard’s similarity (exp) and its SD were estimated based on 10,000 draws (σexp). For each site, PERMDISP was used to test the difference between the observed community similarity (Jobs) and the average of the expected community similarity (exp). Nonsignificant differences (*P* > 0.05) indicated that stochastic processes were the primary driver of community assembly and significantly larger (or smaller) Jobs relative to exp indicates that the deterministic process of ecological filtering (or competitive conclusion) was the primary driver of community assembly.

**References**

Chase JM (2010) Stochastic community assembly causes higher biodiversity in more productive environments. *Science* 328:1388–1391.

Zhang X, Johnston ER, Liu W, Li L, Han X. (2016). Environmental changes affect soil bacterial community primarily by mediating stochastic processes. *Glob Change Biol* 22: 198–207.

Zhang X, Liu W, Bai Y, Zhang G, Han X. (2011). Nitrogen deposition mediates the effects and importance of chance in changing biodiversity. *Mol Ecol* 20: 429–438.

Zhou J, Deng Y, Zhang P, Xue K, Liang Y, Van Nostrand JD, Yang Y, He Z, Wu L, Stahl DA et al. (2014). Stochasticity, succession, and environmental perturbations in a fluidic ecosystem. *Proc Natl Acad Sci USA* 111: E836–E845.
