# Supplementary material for: Rainfall Shapes the Diversity of Soil Nitrogen‐Fixing Microorganisms Worldwide
Source: Adv Sci (Weinh). 2026 Aug 17:e77215. Online ahead of print. doi: 10.1002/advs.77215 (PMC13479853; doi:10.1002/advs.77215)
Supplement: Supplementary file 2 [file ADVS-9999-e77215-s015.docx]

**Fig. S1. Effect of ecosystem type on the relative abundance (a), richness (b), and composition (c) of soil potential N-fixing microbial communities.**

**Fig. S2. Contribution of various climate/plant/soil factors to the composition based on Bray–Curtis distance (a) and unweighted UniFrac distance (b) of soil potential N-fixing microbial communities across different ecosystems.** *, ** and *** represent *P* < 0.05, 0.01 and 0.001, respectively. AI: aridity index; MAP: mean annual precipitation; MAT: mean annual temperature; NDVI: normalized difference vegetation index; Clay+Silt: the clay and silt content in soil; TOC: total organic carbon content; TN: total nitrogen content.

**Fig. S3.** **Effect of MAP on the relative abundance of the six dominant phyla.**

**Fig. S4. The rarefaction curve of the potential N-fixing microbial communities.**

**Fig. S5. Contribution of various climate/plant/soil factors to the difference in the relative abundance (a), richness (c) and** **PCoA axis 1 (e) of soil potential N-fixing microbial communities among various ecosystems (for the 576 samples from each of which 200 reads were randomly drawn), and the linear relationships between the 3 community variables and MAP (b, d, f).** In a, c and e, red and blue represent positive and negative effect, respectively; *, ** and *** represent *P* < 0.05, 0.01 and 0.001, respectively. AI: Aridity Index; MAP: mean annual precipitation; MAT: mean annual temperature; NDVI: normalized difference vegetation index; Clay+Silt: clay and silt content; pH: Potential of Hydrogen; TOC: total organic carbon content; TN: total nitrogen content.

**Fig. S6.** **Contribution of** **various climate/plant/soil factors to the difference in the relative abundance (a), richness (c) and PCoA axis 1 (e) of soil potential N-fixing microbial communities among various biomes for the 120 China samples, and the linear relationships between the three community variables and MAP (b, d, f).** In a, c and e, red and blue represent positive and negative effect, respectively; *, ** and *** represent *P* < 0.05, 0.01 and 0.001, respectively. AI: aridity index; AK: available kalium content; AN: available nitrogen content; AP: available phosphorus content; DOC: dissolved organic carbon content; MAP: mean annual precipitation; MAT: mean annual temperature; NDVI: normalized difference vegetation index; Clay+Silt: clay and silt content; TC: total carbon content; TN: total nitrogen content; TP: total phosphorus content.

**Fig. S7. Effect of ecosystem type on the diversity (a) and composition (b) based on the phylogenetic relationships among these reads of soil potential N-fixing microbial communities.**

**Fig. S8. Contribution of various climate/plant/soil factors to the difference in the diversity (a) and composition (c) based on the phylogenetic relationships among these reads of soil potential N-fixing microbial communities among various ecosystems, and the linear relationships between them and MAP (b, d).** In a and c, red and blue represent positive and negative effect, respectively; *, ** and *** represent *P*< 0.05, 0.01 and 0.001, respectively. AI: aridity index; MAP: mean annual precipitation; MAT: mean annual temperature; NDVI: normalized difference vegetation index; Clay+Silt: clay and silt content; TOC: total organic carbon content; TN: total nitrogen content.

**Fig. S9. Linking the result from the direct-separation method with that from the null model method.**

**Fig. S10.** Leave-one-out cross‑validation results of the XGBoost model for predicting the relative abundances of the soil N‑cycling microbiome.

**Fig. S11. The net effect of each environmental factor on each community variable (the relative abundance, richness, and PCoA axis 1) of the soil potential N-fixing microbiome based on linear mixed model.** MAT: mean annual temperature; NDVI: normalized difference vegetation index; Clay+Silt: clay and silt content; TOC: total organic carbon content; TN: total nitrogen content.
